# Evaluation of a Community-Based Advance Care Planning Campaign for a General Adult Population

**DOI:** 10.1177/10966218251362129

**Published:** 2025-10-27

**Authors:** Peiyuan Zhang, Tiffany C. Erbelding, Glenn E. Schneider, Nikki Highsmith Vernick, John G. Cagle

**Affiliations:** ^1^ University of Alabama School of Social Work, Tuscaloosa, Alabama, USA.; ^2^ Horizon Foundation, Columbia, Maryland, USA.; ^3^ Ignited Strategies, Baltimore, Maryland, USA.; ^4^ University of Maryland School of Social Work, Baltimore, Maryland, USA.

**Keywords:** advance care planning, community-based palliative care, living will

## Abstract

**Background:**

Early advance care planning (ACP) has been widely recommended, but overall uptake remains low. Most efforts to improve formal ACP adoption, namely living will (LW) and health care agent (HCA) documentation, focus on medical settings targeted at older adults with serious or chronic illness, while community-based ACP campaigns are limited.

**Objectives:**

Describe the development and implementation of a community-based campaign (2017–2021) aimed at increasing ACP adoption and evaluating its outcomes at both the community level and across subgroups by race, ethnicity, and age.

**Setting:**

Howard County, Maryland, United States.

**Design:**

Nonexperimental study design.

**Measures:**

A mixed-mode, representative survey of 2000+ county residents was conducted biennially between 2016 and 2021 (one survey was delayed due to COVID-19). Main outcome measures were self-attestation to completing LW and HCA documentation.

**Results:**

In total, 6037 respondents over three years completed the survey. χ^2^ analysis showed that the prevalence of ACP adoption increased overall by over 10% (*p* < 0.001) since 2016. While upward trends in ACP adoption were observed across all racial groups, statistically significant increases were found among only White and Hispanic residents (*p* < 0.001). Logistic regression analysis found that increased odds of ACP adoption were associated with the community campaign for residents ≤65 years of age, White residents, married individuals, and those with a regular health care provider after controlling for confounding factors (e.g., odds ratios = 1.35, *p* < 0.001).

**Conclusions:**

Community-based ACP campaigns can result in increased ACP awareness and adoption in the general adult population, though more work is needed to encourage ACP adoption in diverse communities.

## Key Message

This study describes the implementation and evaluation of a community-based advance care planning (ACP) campaign aimed to improve living will and/or health care agent documentation. At the community level, ACP adoption increased by over 10% since the baseline survey, but future work is needed to improve ACP adoption among residents of color.

## Introduction

Advance care planning (ACP) has been identified as an important mechanism to improve the quality of end-of-life care by guiding health care decision making when individuals can no longer speak for themselves.^1,2^ Living wills (LWs) and Durable Powers of Attorney for Healthcare are core pillars of ACP, alongside conversations with providers and loved ones about end-of-life wishes.^3^ They are both legally recognized documents to respectively record future care wishes at the end of life and designate proxy decision makers (also referred to as health care agents [HCAs]) to make decisions when individuals are no longer able to do so.^3^ ACP allows individuals to uplift the things that matter most to them and to express the values that will allow them to live their lives well until the end.^4^ Planning not only improves communication between patients and clinicians but also improves family experiences by reducing grief and lessening depression after suffering a loss.^5^ It also impacts health care utilization outcomes, resulting in increased hospice and palliative care use and fewer emergency department visits and hospitalizations at the end of life.^1,6–9^

Despite nationwide efforts to facilitate ACP (e.g., 2015 Medicare reimbursement for ACP conversations), the overall uptake of ACP among the U.S. general adult population remains low, with only 29% of adults having documented either an LW or a HCA.^10^ This may be partly explained given that most current ACP interventions focus on medical settings targeted at older adults with serious or chronic illnesses.^11–14^ Decisional capacity can be improved regardless of age or stage of health (especially demonstrated during the COVID-19 pandemic), and proactive ACP engagement is advocated for all adults.^15^ Nevertheless, ACP conversations tend to happen too late.^16^ Significant medical decisions made in crisis situations can be highly complex and are often more influenced by the crisis itself than by a patient’s care goals.^17^ Therefore, community-based ACP interventions and initiatives that bring end-of-life care planning options to a broader audience of the adult population are urgently needed.^18^

However, there is a paucity of community-based ACP interventions.^1^ Among limited ACP community campaigns, Respecting Choices™ is the most notable and best studied.^19^ Community-based initiatives have resulted in increased formal adoption of LWs and HCAs and have triggered conversations about end-of-life care by providing accessible resources to the general population.^19^ But most existing community-based ACP interventions are anchored in primary care settings instead of general community settings.^20,21^

Building upon the existing ACP community model and materials, the Horizon Foundation designed and implemented the Speak(easy) Howard (SEH) campaign to facilitate LW and HCA completion among adult residents living in Howard County, Maryland. This work was particularly aided by the passage of a Maryland law that allowed electronic completion of a LW and HCA without requiring the signature of witnesses (MD. Code Ann., Health General §5–602, 2016). SEH was guided by the socioecological framework^22^ and identified five levels of influence on an individual’s behavior. This multifaceted approach facilitated completion of LWs and HCAs on each level: (1) intrapersonal (e.g., individual), (2) interpersonal (e.g., HCAs and family members), (3) organizational (e.g., community organizations), (4) environmental (e.g., marketing campaign), and (5) policy (e.g., advocacy for legislation permitting electronic LWs and HCAs).

This study evaluated whether the SEH campaign would increase the formal adoption of LWs and HCAs in the general adult population of Howard County, Maryland, USA. Given that ACP can be culturally sensitive and may be embraced differently by people for whom family-centered decision-making is paramount, we also explored the uptake of LWs and HCAs within several racial and ethnic groups.^23,24^ Moreover, it was found that some interventions seemed to be more effective among younger age groups than older groups, despite age being a significant indicator of completing ACP processes.^25^ We further examined the potential benefits of SEH in improving formal ACP by age group. A comprehensive evaluation of SEH in a general community setting by race and age is valuable to gaining insights into community-based ACP intervention design, implementation, and dissemination.

## Methods

### Intervention design and implementation

#### Step 1: Exploring public attitudes, barriers, and facilitators toward ACP to determine the direction for the SEH campaign

The Horizon Foundation conducted eight focus groups consisting of 50 residents over age 18 to explore their attitudes including perceived barriers and facilitators to ACP. Each focus group included eight participants and was facilitated by a professional moderator with a duration of approximately 100 minutes. Through analyzing recorded and transcribed focus group conversations, valuable insights were gained that helped shape the direction and strategies for SEH^26^:

1. Normalizing end-of-life care planning

End-of-life care was a daunting and irrelevant topic for most, especially for those at a younger age who perceived planning for end-of-life care to be too distant to rise high on their priority list. Normalizing end-of-life care planning through gamified educational approaches might overcome emotional barriers to documenting LWs and HCAs.

2. Partnering with community organizations

Participants reported that there was no trusted resource that could reach residents with the message about ACP. A full community mobilization of various organizations (e.g., faith and senior communities) who are naturally trusted messengers for their members was critical.

3. Focusing on naming a HCA

Completing a full LW can be overwhelming, especially for younger individuals who are not yet contemplating end-of-life issues. In contrast, designating an HCA may serve as a more accessible entry point into ACP. These initial learnings led the Horizon Foundation to partner with community organizations and design a multi-component, community-wide ACP campaign.

#### Step 2: Partnering with community organizations, getting buy-in and developing the campaign

**FIG. 1.**
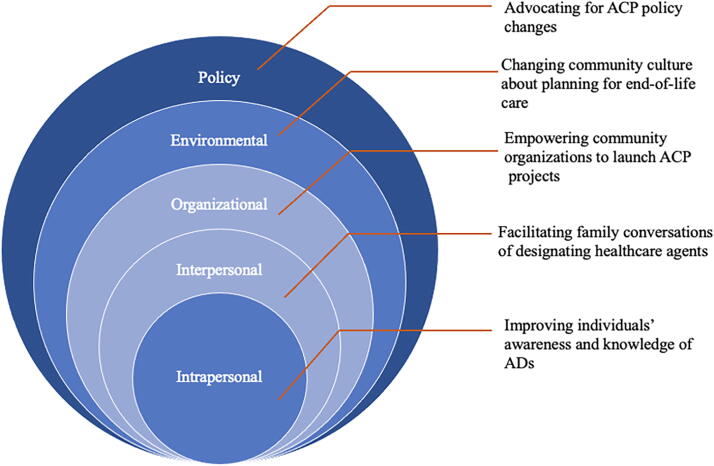
The SEH framework guided by socioecological model. SEH, Speak(easy) Howard.

From Summer 2016 to Summer 2017, the Horizon Foundation partnered with 15 community organizations to promote HCA documentation through brainstorming sessions, monthly calls, and reports. They found that individual-level education was insufficient without institutional support from trusted organizations like churches and senior communities. As a result, SEH worked to improve ACP adoption by (1) improving individuals’ awareness and knowledge of ACP at the intrapersonal level, (2) facilitating conversations about goals of care at the interpersonal level, (3) empowering community organizations to launch ACP-related projects at the community level, (4) changing the community culture about planning for end-of-life care, and (5) advocating for policy changes. The intervention framework was demonstrated by Figure 1.

The campaign used a variety of intrapersonal and interpersonal activities, including film nights and discussions on end-of-life themes at the Columbia Festival of the Arts, interactive games like the HCA spinning wheel, and educational seminars about ACP. Organizational support included infrastructure for electronic ACP adoption, small grants ($500–$5,000) for ACP activities, structured materials, a dedicated facilitator at the local hospital, and integration of ACP into primary care routines. At the environmental level, a marketing campaign with real-life scenarios promoted naming a HCA and documenting care preferences to shift the community’s avoidance of end-of-life planning. The Horizon Foundation also advocated for improved legislative accessibility to ACP. A summary of these interventions is shown in Table 1. More details of the SEH are available in the Supplementary Data.

**Table 1.** Summary of SEH Interventions Across Levels of the Socioecological Model

Levels of campaign	Activities^a^	Descriptions	Time and attendance
Intrapersonal	Arts Events	End-of-life related film screening and post-film discussion, and storytelling events about self-determination and taking control of one’s life and care.	2018 800 attended SEH events at Columbia Festival of the Arts
	Spin the Wheel	A creative and gamified approach to inspire people to think about what their wishes would be and who would make those decisions if they could not speak for themselves. A video recording is available.	2018 Approximately 200 people attended the session
	Get It Done Week	Weeklong educational sessions with one-on-one appointments available for residents to learn about topics including ACP, financial and long-term care insurance planning; caregiving; housing options; health insurance basics; conflict resolution; and estate planning.	2020 Nearly 300 people participated in webinars and over 50 participants received follow up one-on-one help
Interpersonal	Speaking Easy over Dinner	A catered meal delivered with a set of conversation starters. Offered a creative way to encourage residents to talk with their loved ones about the things that would matter most if a time came when they could not speak for themselves.	2020 350 residents participated in Speaking Easy over Dinner
Organizational	Infrastructure: Electronic ads completion website	A website that enabled people to electronically and legally name their health care agent.	2017–present 550 accounts created
	Funding	Small grants given to community organizations who served as trusted messengers and SEH ambassadors. They hosted SEH events and shared SEH messaging through their newsletters and outreach materials.	2017–2021 Funded over 20 organizations with grants between $500 and $5,000 Over 50 community education sessions were held, reaching nearly 2500 residents
	Materials: Structured ACP materials	In collaboration with the Conversation Project of Institute for Healthcare Improvement (IHI), a customizable version of the Conversation Starter Kit for ACP that allowed partners and ambassadors across the community to add their own logos and names. Included a more approachable two-page health care agent designation form (for individuals who prefer a paper form).	2017–present
	Staff: Dedicated ACP facilitator	Funded a full-time clinical social worker at Howard County General Hospital (now Johns Hopkins Howard County Medical Center) to become a dedicated ACP facilitator. Responsible for identifying patients best suited for ACP, initiating ACP conversations, and following up with them to complete ads.	2017–present Over 5000 patients engaged
	Workflow: Embedding in primary care practices	Sponsored three primary care practices to embed “Conversation Ready” principles into daily practices and ensure patient health care wishes could be more comfortably expressed and respected. IHI led a series of webinars, provided one-on-one coaching and conducted site visits at each practice.	2019 While these three practices account for care for thousands of residents, an undetermined number of patients were exposed
Environmental	Marketing campaign	A series of short ads programmed for TV, streaming, and social media platforms were aired over the course of the campaign based on real scenarios and highlighting the importance of naming a HCA and documenting health care preferences.	2017–present 6.6 million impressions (6,635,348) reaching over 600,000 users (621,372) over SEHs lifetime. These numbers indicate that Howard County adults likely saw ads more than once
Policy	Legislation advocacy	Maryland enacted a law allowing electronic LWs and HCAs that do not require the signature of witnesses. Additional legislation passed requiring health care facilities, managed care organizations, and carriers to communicate with patients about ACP. The Horizon Foundation and its community partners advocated for both bills.	2016 2022

^a^ A video summarizing interventions can be found here.

ACP, advance care planning; HCA, health care agent; LW, living will; SEH, Speak(easy) Howard.

#### Step 3: Implementing the campaign

SEH was launched in Howard County in 2017. Between 2017 and 2021, nearly 10,000 different residents attended at least one SEH activity. Over 6 million media impressions were also delivered, ensuring residents likely interacted with at least one SEH advertisement multiple times over the course of the campaign.

### Study design and sample

In partnership with the Howard County Health Department and other organizations, the Horizon Foundation conducted the Howard County Health Assessment Survey (HCHAS) to assess overall health, access to health care services, lifestyle habits, and health knowledge of residents aged 18 years old and above in Howard County.^27^ HCHAS is a biennial survey of a representative sample of adult Howard County, Maryland residents administered through telephone and online panels in 2016, 2018, and 2021 (the 2020 survey administration was delayed due to the COVID-19 pandemic). The survey structure and questions were primarily replicated from the Behavioral Risk Factor Surveillance System,^28^ the nation’s premier system of health-related telephone surveys that collect state data about U.S. residents regarding their health-related risk behaviors, chronic health conditions, and use of preventive services, but HCHAS included questions related to ACP adoption. Approximately 2000 representative respondents were randomly selected from Howard County adult residents to participate in each survey. Once the interviews were collected, statistical weights were applied to the sample to ensure that it was as reflective as possible of the County’s population, according to the most recent data available from the United States Census Bureau. Weights were applied to the following parameters: gender, age, race and ethnicity, and geography. This study was determined to be exempt from review by the University of Maryland Institutional Review Board (#HP-00106902).

This study used data from a total of 6037 completed HCHASs during 2016, 2018, and 2021 to examine at the community level whether (1) there was a significant increase in ACP adoption in 2018 and 2021 after SEH was launched and (2), if so, whether such an increase was associated with the campaign after controlling for demographic characteristics that can impact ACP adoption. SEH was launched widely in 2017 after the 2016 HCHAS. Respondents from the 2016 survey were not exposed to SEH, so they were treated as the baseline.

### Measures

#### Dependent variables

ACP engagement was measured by two items from the HCHAS indicating two different facets of ACP: (1) having a signed LW and (2) having a formal document (either electronic or hard copy) naming a HCA. These were binary response items (0 = No/1 = Yes). “Don’t know/Not sure” or “Refused” responses were treated as missing values.

#### Key independent variable

The “wave” variable was created to indicate different survey years (1 = year 2016, 2 = year 2018, and 3 = year 2021).

#### Covariates

Covariates that may impact ACP engagement were chosen based on the existing studies,^29,30^ including age (years), race (White/Black or African American/Asian/Native Hawaiian or other Pacific Islander/American Indian or Alaska Native), ethnicity (Hispanic or Latino/Not Hispanic or Latino), education status (Never attended school or only attended kindergarten/Elementary School/Some high school/GED or High school graduate/Some college or technical school/College graduate/Graduate level), marital status (Married/Not married), and access to regular health care providers (Yes/No). Education was treated as a continuous variable, with a greater score indicating a higher education degree.

### Analytical analysis

Descriptive statistics were used to summarize the demographic characteristics (i.e., the number and percentage of observations for categorical variables and the mean and standard deviations for continuous variables). Second, χ^2^ analyses between two different aspects of ACP adoption (i.e., LW and HCA) and survey waves were conducted to examine whether there was a significant increase in documenting LWs and HCAs since the intervention was implemented. We further conducted bivariate analyses between ACP adoption and waves, stratified by racial and ethnic groups, to understand changes in LW/HCA adoption prevalence within racial/ethnic subgroups. Finally, given that the likelihood of engaging in ACP can vary significant greatly by the age,^25,30^ we examined whether the observed increase was associated with potential benefits of SEH in both younger and older groups. To do so, we used a commonly acknowledged cutoff point for older adulthood (65 years) to stratify the sample and conducted logistic regression models with subgroup analysis between younger groups and older groups (<65 years vs. ≥65), controlling for demographic characteristics impacting ACP uptake. A two-tailed assumption and alpha threshold of *p* < 0.05 were used to determine statistical significance. Data were analyzed using Stata Version 16.

## Results

A total of 6037 respondents from three waves completed the survey. The mean age of the sample was 51.9 years (standard deviation [*SD*] = 17.3). The unweighted sample comprised of mostly White individuals (*n* = 4058, 70.8%), non-Hispanic respondents (*n* = 5735, 95.5%), and slightly over half were females (*n* = 3154, 52.2%). Less than 20% of the sample were African Americans (*n* = 1031, 18.0%). There were fewer Asians (*n* = 584, 10.2%), Native Hawaiians and other Pacific Islanders (*n* = 26, 0.5%), and American Indians (*n* = 34, 0.6%). The sample sizes from some racial and ethnic populations are not intentionally small—rather, these groups comprise a smaller part of the overall Howard County population, and these numbers reflect the raw, unweighted numbers of those surveyed. The average education level was high school or higher (mean = 4.89, *SD* = 2.25). Most respondents were married (*n* = 3866, 64.5%) and reported having at least one regular health care provider (*n* = 5387, 89.5%). Detailed demographic information can be found in Table 2.

**Table 2.** Summary of Participants’ Characteristics (*N* = 6037)

Characteristic	*N* (%)	M (*SD*)
Wave		
1	2001 (33.1)	
2	2002 (33.2)	
3	2034 (33.7)	
Age		50.9 (18.4)
Sex		
Male	2870 (47.5)	
Female	3154 (52.2)	
Transgender	13 (0.3)	
Race		
White	4058 (67.2)	
Black	1031 (17.1)	
Asian	584 (9.7)	
Hawaiians or Native Pacific Islander	26 (0.4)	
American Indian/Alaska Native	34 (0.6)	
Missing	304 (5.0)	
Ethnicity		
Hispanic or Latino	272 (4.5)	
Non-Hispanic or Latino	5735 (95.0)	
Missing	30 (0.5)	
Education level		4.89 (2.25)
Marital status		
Married	3866 (64.0)	
Not married	2127 (35.2)	
Missing	44 (0.8)	
Having regular health care provider		
Yes	5387 (89.2)	
No	633 (10.5)	
Missing	17 (0.3)	

**FIG. 2.**
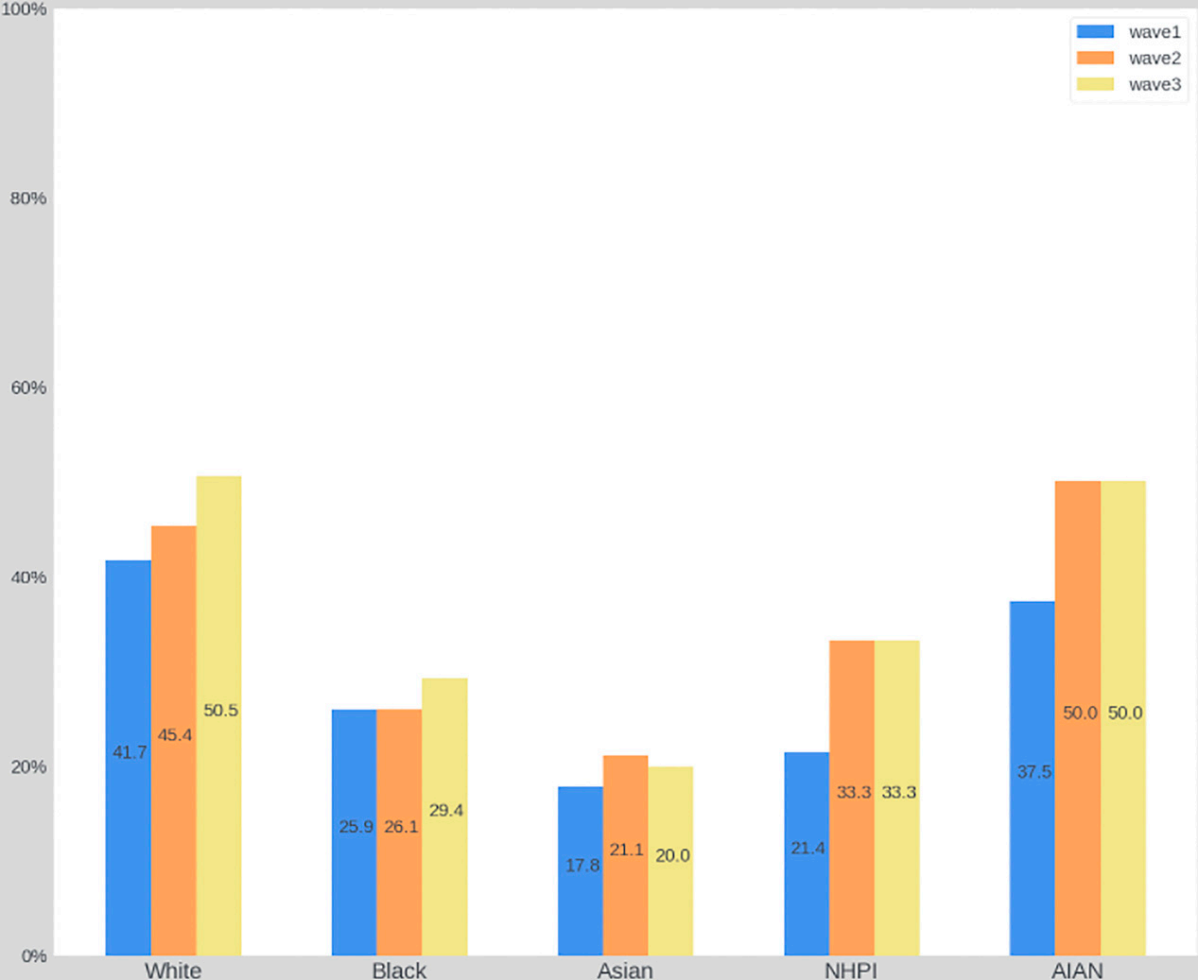
The prevalence of LW from 2016 to 2021 among racial groups. AIAN, American Indian/Alaska Native; LW, living will; NHPI, Native Hawaiian or other Pacific Islander.

**FIG. 3.**
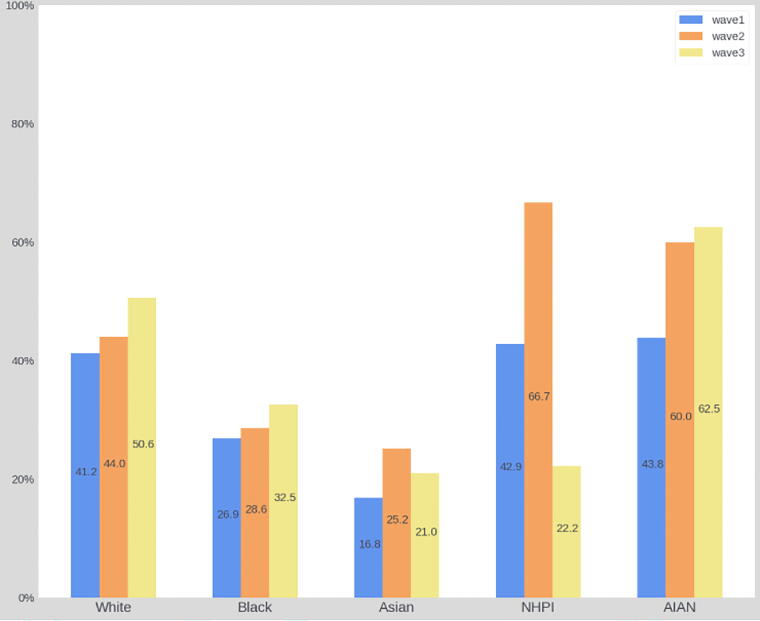
The prevalence of HCA from 2016 to 2021 among racial groups. HCA, health care agent.

**FIG. 4.**
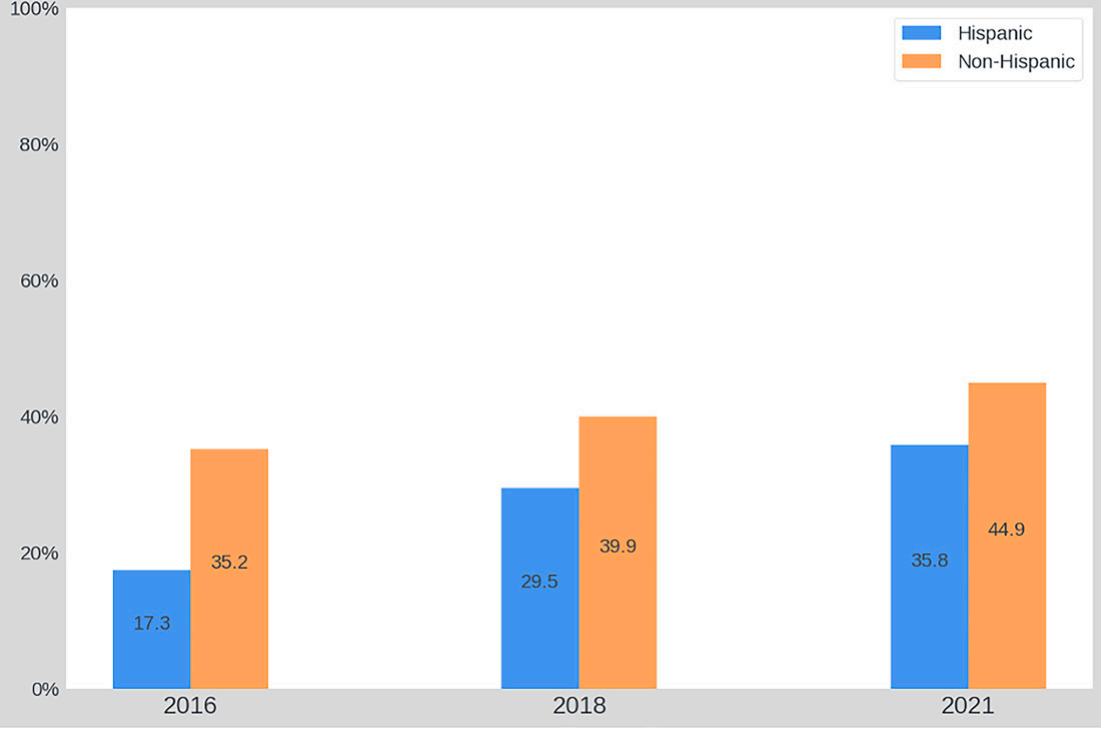
The prevalence of LW from 2016 to 2021 among ethnic groups.

**FIG. 5.**
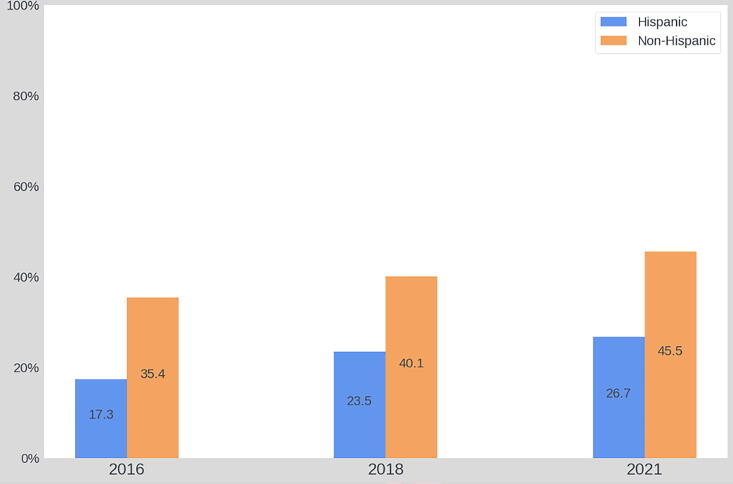
The prevalence of HCA from 2016 to 2021 among ethnic groups.

Overall, at the general community level, formal aspects of ACP engagement significantly increased since the baseline wave in 2016. Specifically, the prevalence of LWs and HCAs increased by over 10%, respectively from 34.3% to 44.6% (χ^2^ = 44.1, *p* < 0.001) and from 34.1% to 45.3% (χ^2^ = 51.9, *p* < 0.001). By conducting bivariate analyses within each racial and ethnic group, we found LW and HCA documentation rates increased across all racial and ethnic groups since 2016. Statistically significant improvements, however, were only found among White participants (LW: χ^2^ = 20.96, *p* < 0.001; HCA: χ^2^ = 25.21, *p* < 0.001) and Hispanic residents (LW: χ^2^ = 8.44, *p* = 0.02; HCA: χ^2^ = 14.8, *p* = 0.001). See Figures 2–5.

Logistic regression results with subgroup analysis found that increased uptake of LWs and HCAs among younger groups (<65 years old) were significantly associated with SEH after controlling for confounding factors (see Table 3). Younger participants at Wave 2 and Wave 3 had respectively 22% and 25% higher odds to have completed LWs (respectively, *p* = 0.03, 95% confidence interval [CI], 1.02, 1.45; *p* = 0.02, 95% CI, 1.03, 1.48) compared with their counterparts in Wave 1 (Model 1). Similarly, Model 2 demonstrated that younger participants at Wave 2 and Wave 3 had, respectively, 22% and 35% higher odds of designating a HCA compared with their counterparts in Wave 1 (respectively, *p* = 0.03, 95% CI, 1.03, 1.44; *p* = 0.001, 95% CI, 1.14, 1.61). Additionally, older adults, White residents, married individuals, and those with a regular health care provider had higher odds of having completed ACP compared with their counterparts (*p* < 0.01).

**Table 3.** Logistic Regression Findings Predicting Advance Directive Completion Stratified by Age Group (*N* = 6037)

	Model 1 (LW)		Model 2 (LW)		Model 3 (HCA)		Model 4 (HCA)	
Groups	Age <65 (*n* = 4066)		Age ≥65 (*n* = 1487)		Age <65 (*n* = 3953)		Age ≥65 (*n* = 1561)	
Variables	OR	95% CI	OR	95% CI	OR	95% CI	OR	95% CI
Wave								
(1)								
2	1.22^*^	(1.02, 1.45)	0.73^*^	(0.55, 0.96)	1.22^*^	(1.03, 1.44)	0.76	(0.61, 1.07)
3	1.25^*^	(1.03, 1.48)	0.96	(0.73, 1.25)	1.35^**^	(1.14, 1.61)	0.88	(0.73, 1.24)
Gender								
(Male)								
Female	1.01	(0.88, 1.17)	1.19	(0.94, 1.50)	1.01	(0.88, 1.16)	1.17	(0.93, 1.48)
Transgender	3.42	(0.77, 15.08)	N/A		2.18	(0.41, 11.67)	N/A	
Race								
(White)								
Black	0.57^**^	(0.47, 0.69)	0.47^**^	(0.35, 0.65)	0.70^**^	(0.58, 0.84)	0.41^**^	(0.30, 0.56)
Asian	0.37^**^	(0.29, 0.48)	0.25^**^	(0.14, 0.45)	0.45^**^	(0.35, 0.57)	0.16^**^	(0.08, 0.31)
NHPI	1.01	(0.38, 2.70)	0.38	(0.02, 6.65)	1.71	(0.72, 4.09)	0.40	(0.03, 6.54)
AIAN	3.70^*^	(1.54, 8.89)	0.24^*^	(0.06, 0.95)	5.58^**^	(2.31, 13.46)	0.27	(0.07, 1.10)
Being non-Hispanic	0.97	(0.64, 1.46)	1.81	(0.57, 5.74)	0.82	(0.55, 1.23)	0.89	(0.31, 2.43)
Education	1.19^**^	(1.11, 1.28)	1.15^**^	(1.07, 1.23)	1.13^**^	(1.06, 1.21)	1.34^**^	(1.22, 1.48)
Being married	2.43^**^	(2.05, 2.88)	1.30	(1.03, 1.65)	1.95^**^	(1.66, 2.30)	1.05	(0.99, 1.59)
Regular health care provider	2.46^**^	(1.88, 3.20)	1.69	(0.97, 2.94)	2.15^**^	(1.67, 2.72)	1.66	(0.96, 2.87)

^*^ *p* < 0.05.

^**^ *p* < 0.01.

AIAN, American Indian/Alaska Native; HCA, health care agent; LW, living will; NHPI, Native Hawaiian or other Pacific Islander.

In summary, the prevalence of ACP adoption at the community level significantly increased compared to 2016 and since the SEH campaign was widely launched in 2017. Further logistic regression analysis controlling for demographic characteristics showed that such increased prevalence was significantly associated with SEH implementation.

## Discussion

This study described SEH—a community-based, multi-level, ACP intervention targeted at the adult population—and examined the prevalence of formal LW and HCA documentation associated with the campaign. Overall, the prevalence of LW adoption (34.3%) at the baseline in Howard County was reflective of the national rate (36.7%).^10^ The prevalence significantly increased by over 10% and reached 44.6% in 2021. Similar trends were demonstrated in HCA documentation as well, increasing from approximately 34.1% to 45.3% from 2016 to 2021.

The community-level significant increase in the prevalence of both LWs and HCAs was not merely a result of an individualized approach but an outcome of community-wide multi-level efforts guided by the socioecological model. At the individual and family levels, both the traditional approach of delivering ACP-related information (e.g., *Get It Done Week*) and a gamified and light-hearted approach to normalizing planning for end-of-life care were used (e.g., *Spin the Wheel* and *Speaking Easy Over Dinner*). Previous studies identified the potential of games in overcoming the reluctance and resistance to discussing end-of-life care. For example, an end-of-life conversation game, “Hello,” consisting of 47 cards with a mixture of light and serious questions that can prompt players to share their values about end-of-life care was implemented in over 50 communities and was found to increase the engagement of community-dwelling adults’ ACP engagement.^31–33^

At the organization level, SEH used a grassroots approach to empower organizations by providing funding, customizable ACP structured materials, and a dedicated ACP facilitator and point of contact in the hospital and primary care settings. These interventions facilitated a cultural shift in beliefs about ACP and supported organizations in conducting ACP activities using approaches they deemed more acceptable to their members. The benefits of the grassroots approach and the collaborative partnership with community organizations were also demonstrated by a recent community-based ACP intervention study in Canada, where researchers partnered with community organizations to develop a training curriculum for ACP.^34^

Additionally, it is worth noting that this community-based multi-level campaign worked particularly well among younger groups under 65 years old. Younger participants in 2018 and 2021 had over 20% higher odds of having completed an LW and HCA compared with their counterparts in 2016 before the campaign was widely launched. This change was especially meaningful as it might be an indicator of early ACP engagement and improved awareness of the public about ACP. This is a long-standing priority in the field of ACP as it allows individuals to have the greatest chance to engage physically and cognitively, at their own pace, and make decisions.^35–37^ However, we failed to observe a significant improvement in formal ACP adoption among older groups. Despite disseminating a series of web-based advertisements based on real scenarios highlighting the importance of documenting a LW and HCA via internet and social media channels, older adults (possibly using technology less frequently) were less likely to be exposed to them. Older adults were more likely to engage when one-on-one support was provided (e.g., through the hospital and primary care facilitators). Future efforts should consider providing additional opportunities for more direct help to increase uptake among older adults.

Finally, our study found that LW and HCA completion rates have increased across all racial and ethnic groups since 2016. Within racial groups, statistically significant improvements were only observed among White and Hispanic participants. This was observed despite intentional outreach and engagement in these communities, including the Horizon Foundation offering a round of targeted funding for organizations with large and diverse populations to provide educational sessions and disseminate information to their membership.^24^ This finding may be explained by persistent and systemic barriers to ACP engagement among racial and ethnic groups, such as mistrust of the health care system^38^ and limited health literacy due to language barriers.^39^ Future efforts should focus on enhancing the cultural awareness of ACP campaigns and addressing structural challenges to better serve diverse communities.^40^

This study had some limitations. Due to limited resources, individual tracking across waves was not possible so increased LW and HCA prevalence at the community level can only be inferred. It was difficult to confirm if respondents were directly exposed to the SEH intervention, as increases may reflect a broader heightened sense of urgency that accompanied the COVID-19 pandemic.^41^

Despite these limitations, this study addresses gaps in community-based ACP interventions for general adults. The significant increase in the adoption of LWs and HCAs from 2016 to 2021 highlights the potential benefits of a multi-level community ACP campaign. Future studies should explore the effectiveness of such interventions with more rigorous designs.

## Abbreviations Used

ACP: Advance care planning
LW: Living will
HCA: Health care agents
