# Supplementary material for: Evaluation of a Community-Based Advance Care Planning Campaign for a General Adult Population
Source: Palliat Med Rep. 2025 Oct 27;6(1):509–20. doi: 10.1177/10966218251362129 (PMC12670688; doi:10.1177/10966218251362129)
Supplement: Supplementary Data [file 10966218251362129_supplementarydata.pdf]

Health Care Agent Initiatives: What Works?

Best Practices for Health Care Providers, Nonprofits & Foundations

As the largest independent health philanthropy in the state of Maryland, the Horizon Foundation has been leading our own health care agent initiative, Speak(easy) Howard, since 2016. Working closely with hospitals, nursing and assisted living facilities, faith communities, nonprofits and other foundations, we have steadily increased the number of health care agents named in our immediate community – Howard County – each year.
Now, we'd like to share lessons learned.

What Is a Health Care Agent Initiative?

A health care agent initiative is an outreach effort aimed at encouraging people in your community to name a health care agent – a person responsible for making decisions in a medical emergency. These initiatives can be key to honoring health care wishes and protecting the quality of life in your community.

Health care agent initiatives also offer big benefits for organizations that lead them. In our own work, we have seen this through continued funding, recognition as an industry leader among Maryland's advance care professional community, and deepened community relations through partnerships with a variety of local organizations.

Benefits of a Health Care Agent Initiative

Health Care Providers

- Improves patient satisfaction scores (which impacts funding, clinical outcomes, patient retention, and medical malpractice claims)
- Reduces likelihood of lawsuits for care that is not congruent with patient wishes
- Improves efficiency and reduces stress for care teams
- Reduces prevalence of unwanted care (and associated costs)
- Allows for reimbursement for planning conversations
- Most importantly, helps honor patient wishes

Nonprofits & Foundations

- Demonstrates measurable impact (number of health care agents named) to funders, donors and board members
- Advances your mission in a new way
- Combats health disparities
- Reduces health care costs in your community

As our foundation expands from primarily working in Howard County to institutionalizing advance care planning and health care agent work across the state, we need more organizations to join the fight.

We know that taking on a new initiative is no small feat. You need initiatives that align with your organization's goals and have a proven track record with others in your space. This overview aims to address both of those pain points. We are grateful to our many partners whose work contributed to the learnings in this resource.

Best Practices for Health Care Providers

- **Focusing initial efforts on patients motivated to act:** People know they won't live forever. We all understand that everyone is at risk of a medical emergency or serious medical diagnosis. Decades of behavioral science research shows that future risk rarely leads to action today. Instead, health care providers are finding success by focusing on the subset of their broader patient audience that is likely motivated to act now. This approach is similar to using the Stages of Change model often referenced in public health. Examples include patients who have recently been diagnosed with a serious or late-stage illness, patients preparing to give birth or have surgery, and patients admitted to the intensive care unit. Some providers even focus on engaging family members of those recently diagnosed with a serious illness as they may eventually take on decision making. Providers can pilot a health care agent initiative with this motivated segment of the population, gather data on the success of those efforts, and use that data to obtain support and resources for an expanded effort that serves more patients in the future.

> **Approach Worth Testing:** In addition to identifying a focal segment, some providers set criteria for identifying patients who fall into that segment upon admittance or during routine check-ins. Your team could consider setting up general criteria or customized criteria by discipline, such as oncology, neurology, obstetrics, etc.

- **Using a multi-component approach:** Systems used by health care providers are often interdependent. That is why providers are seeing results by updating several components of their processes that together improve engagement in health care agent initiatives. Those components vary by provider, but typically include:

  » **Training:** To increase staff understanding of Maryland laws related to advance care planning; to assist staff in pursuing reimbursement for advance care planning conversations; and to increase staff comfortability with advance care planning conversations

  » **Updating workflows:** To identify clear moments when health care agent information will be captured and clear processes for when that information will be accessed

- » **Leveraging electronic health records (EHRs):** To ensure health care agent information is captured in this easily accessible format and to set up a means of accessing EHRs from other provider facilities across Maryland

Well-paced, manageable change is better than none. Many providers begin by taking small, focused action around each of these components and building on their efforts over time.

Using an intervention-style approach: Several studies have validated the success of actively engaging patients in direct conversation about advance care planning behaviors, including the naming of a health care agent.[1] One study found that interventions that relied on material dissemination alone were less effective at changing behavior than facilitated interventions.[2] Health care providers are finding ways to intervene that align with their resources, whether that means identifying a few key touchpoints and staff members to actively engage every patient within a target segment or obtaining funding for a system-wide intervention.

See It In Action: Systemwide Advance Care Planning Intervention

WellSpan Health, a system of eight hospitals and more than 170 outpatient locations, adopted a systemwide approach to implement team-based advance care planning interventions among patients. During the pandemic, they also created a remote response team to help high-risk patients with Covid-19 with advance care planning. The WellSpan team analyzed ICU use and costs for 356 patients who died of Covid-19 after being admitted to hospitals within their system. Patients who had completed ACP prior to hospitalization (53%) were compared with those who had not (47%).[3] While the patients who completed the ACP process were older than those who did not (79 vs. 73 years) and had greater acuity (LACE + score 71 vs. 65; LACE represents a composite score, where L stands for length of stay, A for acuity, C for comorbidities and E for emergency department visits within last 6 months), they were less likely to use the ICU (62% vs. 78%) and accrued 25% lower costs whether they received ICU care or not. This effort serves as an example of how systematic intervention can reduce unwanted medical procedures and the cost of care by effectively understanding and responding to patient wishes.

The work began in 2014, when WellSpan evaluated national and local research highlighting gaps in ACP processes that led to poor outcomes. They realized that their own approach was insufficient because staff focused on documentation and other priorities at the expense of advance care planning conversations. Family members and health system staff were often in conflict and clinicians lacked training on engaging patients and family members around the subject.

WellSpan documented the intervention approach that generated improved outcomes (see below). You can read more about this approach at https://catalyst.nejm.org/doi/full/10.1056/

[1] Ramsaroop, S.D., Reid, M.C. and Adelman, R.D. (2007), Completing an Advance Directive in the Primary Care Setting: What Do We Need for Success? Journal of the American Geriatrics Society, 55: 277-283. https://doi.org/10.1111/j.1532-5415.2007.01065.x

[2] Diegelmann, S., Bidmon, S., & Terlutter, R. (2022). Promoting advance care planning via Mediated Health Resources: A Systematic Mixed Studies Review. Patient Education and Counseling, 105(1), 15–29. https://doi.org/10.1016/j.pec.2021.06.0

[3] Vipul Bhatia, M. D., Roberta Geidner, M. A., Kamna Mirchandani, M. S., Yue Huang, M. S., & Haider J. Warraich, M. D. (2021, August 18). Systemwide advance care planning during the COVID-19 pandemic: The impact on patient outcomes and cost. NEJM Catalyst Innovations in Care Delivery. Retrieved August 10, 2022, from https://catalyst.nejm.org/doi/full/10.1056/CAT.21.0188

Horizon Planning Workflow for Primary Care Practice

Horizon planning workflow for primary care practice at WellSpan Health (WSH). The systemwide advance care planning (ACP) intervention was developed by a task force supported by WellSpan's board of directors and executive leadership. By 2016, WellSpan had implemented a pilot with 10 primary care practices to test the model illustrated. APP = advanced practice provider, DPOA-HC = durable power of attorney for health care, EOL = end of life, POA = power of attorney, POLST = physician's orders for life-sustaining treatment, SW = social worker.

WELLSPAN HEALTH

Day of Patient Visit

CLINICIAN

- Selects patients who do not have a Living Will on file. Prioritize high-risk patients who may be susceptible to Covid-19.
- Confirms patient selection with physician/APP before visit.
- Introduces Horizon Planning® while rooming the patient.
- Provides patient with Horizon Planning packet.
- Completes Advance Care Planning (ACP) Note in Epic to document patient. conversation and outcome.
- Enters Health Care Agent information in EPIC if patient has a DPOA-HC document.

PHYSICIAN / APP

- Discusses Horizon Planning® with patient and gives them:
  - Horizon Planning® brochure,
  - Horizon Planning® Patient Instructions,
  - Advance Care Planning Step-by-Step Guide,
  - Five Wishes® booklet, and
  - Patient Education Guide on End-of-Life Treatments.
- **Send Ambulatory Referral to Care Coordination for Horizon Planning.** Indicate Phase of Life 1-4 for patient in ACP note.
- Documents with an ACP note in EPIC conversation with patient. Can use Dragon mike. **Smart Link** dot phrase **".acpbegin"**, text regarding your conversation with patient, then **".acpend"**.
- Create POLST and Palliative or Hospice Care Referral when patient in last year of life. Use **EOL Index.**

1-2 Weeks Later

CHRONIC CARE COACH/ AMBULATORY SW/ OTHER CLINICAL STAFF

- **Care Coordination staff** will select appropriate staff for follow-up referral for Horizon Planning.
- Staff member selected contacts Patient up to three times for follow-up.
- Completes ACP Note in Epic.
- Completes Phase of Life 1-4 templates depending on phase established by physician/APC.
- Requests patient to complete and return the Five Wishes® booklet.
- Sends referral to **WSH Horizon Planning Pool** if needs more in-depth facilitated conversation and assistance in completing documents directly.

At a Later Date

PATIENT RETURNS THE FIVE WISHES® BOOKLET

- POA sends copy of Five Wishes® to Clinical Staff for review and approval before scanning.
- OR checks that Living Will is signed and dated, and DPOA is signed and dated. Both have two witnesses. Durable Power of Attorney cannot witness document.
- POA/Medical Assistant follow workflow for scanning Advance Directives.
- Enters Health Care Agent information in EPIC if patient has a DPOA-HC document.

Once the Horizon Planning® ACP Note is complete, it is considered that you have completed this portion of the Horizon Plan®.

Source: WellSpan

NEJM Catalyst (catalyst.nejm.org) © Massachusetts Medical Society

See It In Action: Communitywide Advance Care Planning Interventions
Since 1986, Gundersen Lutheran Health System, based in La Crosse, Wisconsin, has leveraged healthcare providers and community leaders to increase both the completion and accessibility of advance care plans in their community. Their efforts have been astoundingly successful. The health system is ranked as one of the top ten hospitals nationwide, and in 2017, 96% of all La Crosse residents who died the year prior had completed advance directives, far exceeding the national average.

The nationally renowned work began with one man asking for change. Dr. Bud Hammes, a medical ethicist at Gundersen, convinced hospital administrators in the late 1980s that end-of-life care needed to be given higher priority. They began by creating an electronic medical record system to document patient preferences. They also made end-of-life conversations, disease specific planning and advance directives part of the hospital's routine practice.

Dr. Hammes says the secret to the effort's success is that they look beyond the hospital and call on community leaders – trained as advance care planning "facilitators" – to regularly engage people outside of healthcare settings. In an interview with KALW Public Media, Hammes said, "It isn't just about training a few people to have these conversations with patients.[4] It's actually about changing the culture of the health care system and ultimately of the community." The system aims to have enough touchpoints throughout the community that people are regularly triggered to consider advance care planning in their everyday lives, and if they do become a hospital patient, a system is in place to close the loop and ensure that planning is completed. Some community members who are not even patients come to Gundersen to seek advance care planning counseling from the hospital's facilitator.

Gundersen Health now trains other hospital systems on the pillars of successful intervention design through their not-for-profit organization called Respecting Choices. Learn more at https://respectingchoices.org/.

- **Using EHRs to capture and access health care agent information:** The benefit of capturing health care agent information within EHRs is well established: It is easier for providers to find this information if it is embedded in the recording system they already use. Other forms of sharing health care agent information can fall woefully short. One study of advance directive behavior found the following: Of patients under age 75 who simply communicated information about their health care agent to their provider (but did not provide documentation), agent contact information was available in the medical record 0% of the time.[5] Providers are finding success in using health care agent information with EHRs by focusing on: (1) planning what triggers capturing a patient's health care agent information within EHRs, (2) determining which staff members are responsible for capturing and accessing that information, and (3) connecting to state-wide databases (like CRISP, Maryland's health information exchange) that share this critical information across providers and health care systems.

[4] Mar, J. A. (2017, January 24). How do you want to die? Write it down. KALW.
[5] Committee on Approaching Death: Addressing Key End of Life Issues; Institute of Medicine. Dying in America: Improving Quality and Honoring Individual Preferences Near the End of Life. Washington (DC): National Academies Press (US); 2015 Mar 19. PMID: 25927121.

Trends in Increasing Integration of Health Care Agent Information and EHRs:

- Some EHR systems have implemented a dedicated advance directive tab in patients' records, which makes the information easy to find in an emergency.
- Several hospitals and health care systems are putting an indication on a record's main page if the patient has end-of-life documents.
- Private companies, like MyDirectives, are launching websites that allow patients to complete and upload their health care agent forms so that their doctors can access them when needed. ADVault, the firm that operates MyDirectives, has been approved by the Maryland Health Care Commission as an electronic advance directives vendor in the state. It is also the only electronic advance directives service approved to connect to CRISP, allowing providers across the state to access completed advance care plans.

Resource Spotlight: CRISP

Using a statewide database like CRISP can help providers instantly access existing patient advance directives through any Internet browser. As the designated Health Information Exchange (HIE) in Maryland and D.C., it is free to all ambulatory practices and includes clinical information from all 50 acute care hospitals in Maryland and all 7 in D.C. Learn more about CRISP: https://www.crisphealth.org/

Identifying a care coordinator: Many health care providers have bolstered their health care agent initiatives by assigning one or more staff members to play the critical role of care coordinator – the person who acts as the throughline for patients engaged in these initiatives. For providers with the resources to have one person whose time is fully devoted to this role, often coordinators are involved from beginning to end. They may be the person who initially engages patients in health care agent conversations, walks them through the naming process, and follows up to ensure that agent's information is captured. For providers with limited resources for such a role, a coordinator may simply be assigned to follow through with patients who have been engaged at some other point during their care. As such, some providers request funding for a full-time employee to take on this role; others divvy up the work among several individuals or find manageable ways to work these tasks into the responsibilities of one individual.

See It In Action:
In 2017, Howard County General Hospital (HCGH), part of the Johns Hopkins network, piloted integrating a health care agent coordinator role within their system. Initially funded by the Horizon Foundation, the coordinator was a full time employee dedicated to engaging patients in advance care planning. The coordinator worked with physicians to identify patients best suited for advance care planning, visited those patients to understand their concerns and share initial information. And most critically, the coordinator was charged with following up with patients to ensure their advance directives were completed. With the help of this position, the hospital has captured over 5,000 advance directives.

- **Engaging patients continually, over time:** Although naming a health care agent can be one of the less burdensome advance care planning behaviors, the process can still take time. Health care providers are planning for this by embedding multiple opportunities to discuss health care agents within their workflows and relying on those playing the care coordinator role to follow up. Some providers have seen success by setting a number of follow-up engagements per patient and even following up after patients have been released from care.

Best Practices for Nonprofits

It is logical to assume that people are most likely to engage in advance care planning activities while interacting with health care providers. However, research and experts in the field often challenge that assumption. In fact, Respecting Choices, a nonprofit born out of the Gundersen Health System and renowned as a leader in creating effective, evidence-based advance care planning initiatives, cautions against over-relying on health care providers and encourages engaging community organizations in this work. Why? Behaviors like naming a health care agent are personal, and often require reflection, guidance and time. As deeply connected, trusted entities within communities, nonprofits are uniquely positioned to play a critical role in encouraging communities to engage in health care agent initiatives.

- **Using a specified focus to reach population segments that experience disparities in advance care planning:** Communities of color – namely, Hispanic, Asian American and African American communities – often lag behind their white counterparts in terms of advance care planning behaviors. Additionally, the unique needs of people with intellectual disabilities or those facing Alzheimer's are sometimes unaccounted for in advance care planning initiatives.[6] Nonprofits, which often focus on specific population

[6] Committee on Approaching Death: Addressing Key End of Life Issues; Institute of Medicine. Dying in America: Improving Quality and Honoring Individual Preferences Near the End of Life. Washington (DC): National Academies Press (US); 2015 Mar 19. PMID: 25927121

segments in their broader work, are stepping in and using their specialized expertise to engage these segments in health care agent initiatives. This allows nonprofits to advance their missions in new ways while making measurable impact in spaces where others cannot.

See It In Action:
In Columbia, Maryland, St. John the Evangelist Roman Catholic Church has successfully engaged hundreds of parishioners in advance care planning activities for years. Priests have discussed advance care planning from the pulpit, volunteers have organized conversation workshops and the church has worked closely with the Horizon Foundation's Speak(easy) Howard initiative as a community partner.

Work began when the volunteer church committee researched Catholic teachings on advance care planning, met with the Archdiocese of Baltimore, mapped out their outreach and worked with the four priests at St. John the Evangelist to provide resources and information. Priests began sharing information by delivering homilies on advance care planning during mass. Each priest uniquely described how important end-of-life decisions were to them and used their own personal stories to urge parishioners to take important steps in planning for their own care.

In addition to the message coming from the pulpit, church members heard about Speak(easy) Howard in numerous ways. An initiative committee member attended each mass to answer questions. Newsletters and emails included notices. Workshops took place immediately after mass. Conversation tools were distributed to show people how to begin their discussions and name their health care agents online. In addition, the church held three conversation workshops with as many as 120 people at each session. Panel discussions that answered questions about the legal and medical components of advance care planning had more than 300 attendees.

St. John the Evangelist also made a concerted effort to ensure the message reached the church's Spanish-speaking members who make up nearly half the congregation. One of the church's associate pastors delivered Spanish homilies that emphasized the importance of determining and discussing these decisions now.

The church is continuing to expand its efforts with regular events and resource offerings. A cultural shift has occurred among parishioners. All in all, fully committing to advance care planning outreach made an impact in their church and overall community.

+ **Acting as convener:** Because of their ability to leverage community connections, nonprofits are also seeing success in playing the role of convener – creating opportunities for communities to come together and discuss, obtain information, and receive assistance around naming a health care agent. Convening can take many forms: workshops that offer personal help in naming a health care agent or talking to loved ones about care wishes, hosting Q&A sessions with experts, and even movie screenings that inspire action. Some nonprofits rally their communities to attend such events hosted by other organizations.

- **Advocating for legislation:** Nonprofits actively rally support for legislation that makes it easier to name a health care agent. Their deep community ties make them especially skilled at this task.

Best Practices for Foundations

Often equipped with the resources, connections and influence to spark change, foundations can significantly amplify interest in health care agent initiatives. Their biggest strength tends to be the ability to combine their ambitious visions for community change with an ability and willingness to support others in the community working towards similar goals.

- **Providing funding to amplify efforts of those already working on health care agent initiatives:** Foundations are seeing success offering grants to nonprofits in support of promising or effective work that they would like to help scale. In particular, grants to nonprofit organizations that are faith-based, health or aging focused, or serve those experiencing disparities in advance care planning have proved fruitful for foundations.

- **Funding research:** Supporting research on advance care planning and health care agent initiatives in particular not only provides valuable insight that allows foundations to better serve their communities but also acts as a baseline by which they can measure success and obtain future funding to support expanding their efforts. Organizations like the Horizon Foundation are focusing current research goals around behaviors that are measurable and comparable to data from previous years, such as the number of residents with a named health care agent or advance directive. They then identify additional insights that relate to their goals, such as differences among people of varying socioeconomic statuses or racial and ethnic backgrounds.

Addressing a Need: Equitable Representation in Advance Care Planning Research
According to the Maryland State Advisory Council on Quality Care at the End of Life, most advance directives are completed by white, female older adults.[7] This segment of the population is also most likely to be included in advance care research and, consequently, is heavily included in research conducted to date. Foundations with goals of increasing health equity may choose to begin their health care agent initiative work by funding studies uncovering barriers experienced by men, younger people, communities of color and people with disabilities to help make their representation in advance directive studies more proportional to their populations.

[7] State Advisory Council on Quality Care at the End of Life. (2020). (rep.). State Policy Recommendations to Increase Electronic Advance Directive Registrations: A Report to the Joint Committee Chairs.

Approach Worth Testing:
Foundations interested in funding research around advance care planning disparities should consider community-based participatory research (CBPR). CBPR takes a partnership approach to research that equitably involves community members and researchers in all aspects of the research process. It enables all partners to contribute their expertise, enhances understanding and encourages the integration of knowledge gained and action to improve community outcomes.

Deep seated barriers to advance care planning in your community can be based in systemic disparities, cultural nuances, religious customs and many other things. Using a CBPR approach allows a community to investigate these barriers together and can result in partnerships with trusted community messengers for implementation, if you decide to create a health care proxy campaign in the future.

Advocating for legislation: Foundations often take a multipronged approach to legislation aimed at increasing the ease of naming a health care agent. This may mean leveraging relationships with local leaders and other entities, creating coalitions with like-minded organizations to amplify influence, or funding campaigns engaging the general public in supporting the issue at hand.

Trends in Advance Care Planning Related Advocacy
Foundations and other organizations working on advance care planning are prioritizing legislative changes that stand to have the greatest impact. These include:

- **Supporting continuing education for health care providers:** Through legislation, states (which oversee medical licensure requirements that include continuing medical education) can ensure that physician education includes training on advance care planning, including health care agent naming, with patients.
- **Supporting standards for electronic health records:** Ideally, all electronic health records platforms would be required by the state to have the ability to proactively search a registry, a health information exchange, or a service like MyDirectives to locate a person’s care plan, advance directive (including their health care agent). It would also be ideal if providers were required to link to a patient’s advance directive in their electronic health record.
- **Incentivizing insurance companies to offer advance care planning opportunities:** This may include offering advance care planning as a covered benefit and/or presenting opportunities to complete an advance care plan during open enrollment.

- **Advocating for systems change:** Foundations are seeing success working to normalize actions that systematically and regularly present individuals in the community with opportunities to name a health care agent. Examples include working with the motor vehicle administration to encourage health care agent declaration during registration and organ donation opt-in and partnering with insurance firms to include naming a health care agent in the annual process of selecting insurance benefits.

- **Creating multi-component initiatives:** Several foundations, especially those focused on health and aging, have reserved funding to develop multi-component, multi-year health care agent initiatives in their communities. These initiatives take on various forms depending on available funding and staff resources. Generally, it is best practice to include strategies that: engage individuals in changing personal behavior; communities in amplifying existing or new efforts; organizations in expanding their influence; and state and local institutions in systematically and regularly presenting the opportunity to name a health care agent.

See It In Action: Speak(easy) Howard Campaign

In 2016, the Horizon Foundation launched the Speak(easy) Howard campaign as a multi-component advance care planning initiative. Since that time, the number of health care agents named in the immediate county serviced by the campaign has steadily increased each year.

We are sharing some of the tactics used in the Speak(easy) Howard campaign below and the areas the campaign seeks to impact. Our hope is that this sparks ideas for other foundations looking to impact advance care planning in a comprehensive, collaborative way.

- **Individuals:**
  - » Designed a public campaign website where residents could name a health care agent online (through a partnership with private firm MyDirectives) and get other resources.
  - » Launched a behavior change marketing campaign targeting adults in the county.
  - » Hosted community events:
    - Stoop Stories: Storytelling event in which performers shared experiences of designing life on their terms. Attendees could name an agent after the event.
    - Values Workshop: Attendees were guided through several activities that helped them consider their values related to quality of life.
    - Speaking Easy Over Dinner: The Foundation sent free meals to families that came with conversation prompts for each course. The prompts encouraged families to consider and discuss their values.
    - Get It Done Day: Virtual event in which residents received one-on-one counseling around a variety of tasks often categorized as "getting one's affairs in order" – will planning, insurance coverage, assisted living, and advance care planning.
- **Communities:**
  - » Designed a two-tier partnership program. Organizations could elect to join the Junior Partner Program, which was not grant funded and asked participants to publicize campaign events and share educational materials. Alternatively, organizations could join the Senior Partner Program, which provided grants up to $5,000 for organizations to publicize campaign events, share educational materials, hold two events per year, and design a system for their own team members to complete advance care plans. To date, the Foundation has worked with over two dozen senior partners.
  - » Provided grant funding to several faith-based and cultural organizations (serving African American, Hispanic American, and Korean American communities) for hosting advance care planning events and distributing educational material.
  - » Funded research through community groups to better understand disparities in advance care planning behaviors among certain racial/ethnic groups.

(continued on next page)

- **Organizations:**
  - Helped fund the installation of a full time coordinator at Howard County General Hospital, who engaged high priority patients in advance care planning during and after a hospital stay.
  - Funded a partnership between the Maryland Hospital Association and the Maryland Healthcare Education Institute to design guidance for hospitals looking to increase advance care planning among patients and expand access of existing advance care planning documents.
- **Systems:**
  - Helped create the Maryland Honoring Choices Coalition - a group that advocates for providing every Maryland resident with regular opportunities to complete an advance care plan.
  - Advised the Maryland Health Care Commission on advance care planning-related legislation during the General Assembly.

The combined efforts of all involved in the Speak(easy) Howard campaign have led to noteworthy successes, including:

- **Statistically significant increases in named health care agents:** The 2021 Howard County Health Assessment Survey (HCHAS) showed statistically significant increases in named health care agents in the County (including increases in most communities of color). From 2016 to 2021, the percentage of named agents increased from 30% to 37% overall. Among racial/ethnic groups, that percentage increased from 37% to 45% for White residents; from 22% to 32% among Black residents; from 16% to 19% among Asian residents; and from 15% to 41% among Hispanic residents.
- **Collaboration with the Institute for Healthcare Improvement (IHI):** In 2016, IHI was contracted to host a community-led learning collaborative. IHI coached the 15 participating organizations through embedding advance care planning into their standard operations.
- **Legislative advances:**
  - HB 1385 (2016): This bill created a secure path for completing electronic advance directives, removing the requirement for paper documents and two witnesses. The bill allowed the Horizon Foundation to create a platform for residents to – in real time – electronically name their health care agents. The foundation played a key part in the bill's passage.

- HB 1073 (2022): Requiring the Maryland Health Care Commission to coordinate the accessibility of electronic advance care planning documents in the State, this bill stands to significantly increase ease in advance care planning activities. The bill also requires certain health care facilities, managed care organizations, and carriers to take certain actions relating to electronic advance care planning documents. The Horizon Foundation, its partners within the Maryland Honoring Choices Coalition, and many other community members were directly involved in the conception of this bill and rallying for its passage.
- SB 837: Maryland's Quality of Care at the End of Life Council created a report of recommendations for embedding advance care planning statewide. Those recommendations were crafted into SB 837, introduced in the 2021 session. Though it passed in the Senate, the bill stalled in the House. The Foundation is working with partners to pursue legislation for 2022.

You've Learned What's Working. Now Let's Make It Work For You.

This best practices report was designed to share what's working for those looking to make an impact in the advance care planning space. Now, we hope you'll put these lessons to work. The Horizon Foundation is grateful for organizations leading the charge to help everyone ensure their wishes are honored.

Please connect with us as you start this journey: join our newsletter on our Speak(easy) Howard homepage or email info@speakeasyhoward.org with any questions.

Resources

General Resources

A Guide to Maryland Law on Healthcare Decisions: Offers a brief overview of Maryland's Health Care Decisions Act, frequently asked questions about advance directives in Maryland, and includes an optional advance directive form from the State. Note: This paper form is optional. You can also center your health care agent initiative around an electronic advance directive, like the Horizon Foundation did with MyDirectives.

Respecting Choices: Offers training for hospital systems and community organizations based on the renowned advance care planning work within the Gundersen Health System.

Speak(easy) Howard Partner Resource Page: Offers free resources for those looking to share educational materials and hosting events in their communities.

Maryland State Policy Recommendations to Increase Electronic Advance Directive Registrations: Recommendations from the State Advisory Council on Quality Care at the End of Life to support electronic advance directive registrations.

Healthcare Provider Resources:

Gaining Traction: Increasing Value and Support for your Advance Care Planning Program: Resource from the Five Wishes Best Practice Community on obtaining support for advance care planning initiatives within hospital systems.

Center to Advance Palliative Care Online Clinical Trainings: Offers several free trainings (for member organizations) aimed at helping physicians build basic advance care planning skills.

Maryland Hospital Association Guide on Effective Advance Care Planning Processes: Details key steps in increasing patient completion and provider accessibility of advance care planning documents within a hospital setting.

"Conversation Ready": A Framework for Improving End-of-Life Care (Second Edition): White paper from the Institute for Healthcare Improvement that offers guidance for health care organizations and clinicians on providing respectful end-of-life care that is concordant with patients' values and goals.

"To Bill or Not to Bill, that is the Question?!" Respecting Choices presentation deck on using advance care planning billing codes in health care daily practices.

Nonprofit and Foundation Resources:

Getting Started Guide for Communities: This guide helps community groups plan advance care planning outreach campaigns with a special focus on encouraging conversations about wishes. It is authored by The Conversation Project, an initiative working to promote advance care planning conversations and documentation.

Community Outreach Tools: The following list features several engaging activities that can be used during community events or as tools as part of a broader campaign.

- The “Hello” Game https://commonpractice.com/
- The Conversation Project http://theconversationproject.org/
- Five Wishes https://www.agingwithdignity.org/
- Go Wish https://codaalliance.org/go-wish/
- Death Deck https://thedeathdeck.com/
- Prepare for Your Care https://www.prepareforyourcare.org/#/pamphlet
- Being Mortal documentary http://www.pbs.org/wgbh/frontline/film/being-mortal/

References

References

1. Diegelmann, S., Bidmon, S., & Terlutter, R. (2022). Promoting advance care planning via Mediated Health Resources: A Systematic Mixed Studies Review. Patient Education and Counseling, 105(1), 15–29. https://doi.org/10.1016/j.pec.2021.06.008

2. Hammes, B.J., Rooney, B.L. and Gundrum, J.D. (2010), A Comparative, Retrospective, Observational Study of the Prevalence, Availability, and Specificity of Advance Care Plans in a County that Implemented an Advance Care Planning Microsystem. Journal of the American Geriatrics Society, 58: 1249-1255. https://doi.org/10.1111/j.1532-5415.2010.02956.x

3. Houben, C. H. M., Spruit, M. A., Groenen, M. T. J., Wouters, E. F. M., & Janssen, D. J. A. (2014). Efficacy of Advance Care Planning: A systematic review and meta-analysis. Journal of the American Medical Directors Association, 15(7), 477–489. https://doi.org/10.1016/j.jamda.2014.01.008

4. Committee on Approaching Death: Addressing Key End of Life Issues; Institute of Medicine. Dying in America: Improving Quality and Honoring Individual Preferences Near the End of Life. Washington (DC): National Academies Press (US); 2015 Mar 19. PMID: 25927121.

5. Maryland Hospital Association. “Advance Care Plan Process.”

6. Mar, J. A. (2017, January 24). How do you want to die? Write it down. KALW.

7. National Academies of Sciences, Engineering, and Medicine. 2021. The challenges and opportunities of advance care planning: Proceedings of a workshop. Washington, DC: The National Academies Press. https://doi.org/10.17226/26119

8. Person centered care: Advance care planning: Shared decision making. Respecting Choices. (2022, July 22). Retrieved August 10, 2022, from https://respectingchoices.org

9. Ramsaroop, S.D., Reid, M.C. and Adelman, R.D. (2007), Completing an Advance Directive in the Primary Care Setting: What Do We Need for Success?. Journal of the American Geriatrics Society, 55: 277-283. https://doi.org/10.1111/j.1532-5415.2007.01065.x

10. State Advisory Council on Quality Care at the End of Life. (2020). (rep.). State Policy Recommendations to Increase Electronic Advance Directive Registrations: A Report to the Joint Committee Chairs.

11. Sokol-Hessner L, Zambeaux A, Little K, Macy L, Lally K, McCutcheon Adams K. “Conversation Ready”: A Framework for Improving End-of-Life Care (Second Edition). IHI White Paper. Boston, Massachusetts: Institute for Healthcare Improvement; 2019.

12. Van Wert, R., & Wallace, E. (2018). Impact of advance care planning interventions on patient and family satisfaction: A systematic review and Descriptive Analysis (S777). Journal of Pain and Symptom Management, 55(2), 698–699. https://doi.org/10.1016/j.jpainsymman.2017.12.431

13. Vipul Bhatia, M. D., Roberta Geidner, M. A., Kamna Mirchandani, M. S., Yue Huang, M. S., & Haider J. Warraich, M. D. (2021, August 18). Systemwide advance care planning during the COVID-19 pandemic: The impact on patient outcomes and cost. NEJM Catalyst Innovations in Care Delivery. Retrieved August 10, 2022, from https://catalyst.nejm.org/doi/full/10.1056/CAT.21.0188
